# Association between cataract and fatty liver diseases from a nationwide cross-sectional study in South Korea

**DOI:** 10.1038/s41598-023-50582-7

**Published:** 2024-01-02

**Authors:** Kyoung Hae Kang, Daeun Shin, Ik Hee Ryu, Jin Kuk Kim, In Sik Lee, Kyungmin Koh, Tae Keun Yoo

**Affiliations:** 1 Cornea, Cataract and Refractive Surgery Division, Department of Ophthalmology, Kim’s Eye Hospital, Konyang University College of Medicine, 136 Yeongshinro, Youngdeungpogu, Seoul, 07301 Republic of Korea; 2 Department of Refractive Surgery, B&VIIT Eye Center, B2 GT Tower, 1317-23 Seocho-Dong, Seocho-Gu, Seoul, Republic of Korea; 3 Research and development department, VISUWORKS, Seoul, South Korea

**Keywords:** Disease prevention, Endocrine system and metabolic diseases, Eye diseases

## Abstract

This study examined the link between fatty liver disease (FLD) and cataracts, as previous research has suggested that FLD may contribute to metabolic syndrome, systemic inflammation, and potentially cataracts. We studied a nationwide cross-sectional cohort of the Fifth Korean National Health and Nutrition Examination Survey 2010–2011. FLD was defined as nonalcoholic FLD (NAFLD) and metabolic dysfunction-associated FLD (MAFLD). Multinomial logistic regression was utilized to investigate the relationship between cataracts and FLD after adjustment for potential confounders. Participants with cataracts had higher liver fibrosis scores, including the NAFLD fibrosis score (NFS; *P* < 0.001), fibrosis-4 index (FIB4; *P* < 0.001), and fatty liver index (FLI; *P* = 0.001). NAFLD was not associated with a higher odds ratio (OR) for cataracts in the fully adjusted model (OR = 1.23, *P* = 0.058). MAFLD was significantly associated with a higher OR (OR = 1.34, *P* = 0.006). After adjusting for all factors, the severity of FLD was linked to an increased risk of cataracts, with significant linear trends (*P* values for linear trends of NFS, FIB4, and FLI < 0.05). After adjusting for well-known cataract risk factors, MAFLD was significantly associated with cataracts. Our analysis suggests that FLD may serve as an independent risk factor for cataracts.

## Introduction

A cataract is defined as the opacification of the crystalline lens in the eye. It is a major pathological ocular disease that causes poor vision and blindness worldwide. Recent studies have demonstrated that systemic metabolic and inflammatory diseases are closely associated with cataract formation. Diabetes is a known risk factor for cataracts^1^, as hyperglycemia can worsen cataract formation by affecting blood vessels and aqueous humor. Hypertension and dyslipidemia are also linked to cataracts^2^. Oxidative stress caused by systemic inflammation is another risk factor. Cataract prevalence increases in inflammatory conditions, such as increased plasma C-reactive protein^3^, celiac disease^4^, and allergic diseases^5^. However, the impact of metabolic disorders or inflammation caused by liver disease on the eyes have not been studied.

Fatty liver disease (FLD) is widespread globally and is a leading cause of liver-related morbidity and mortality^6^. The prevalence ranges from 15 to 30% in East Asian countries^7^. FLD is associated with an increased risk of cardiovascular events, cirrhosis, and liver cancer. This burden increases as the prevalence of metabolic diseases continues to rise. Nonalcoholic FLD (NAFLD) refers to the pathological conditions of hepatic steatosis in individuals who consume limited to no alcohol and do not have a secondary cause of hepatic steatosis, such as viral or drug-induced hepatitis^8^. However, both alcohol intake and metabolic dysfunction should be considered as risk factors when diagnosing FLD. Recently, a novel term, metabolic dysfunction-associated FLD (MAFLD), has been used to focus on liver-related metabolic syndromes among patients with FLD^9^. MAFLD describes the current understanding of FLD related to metabolic syndrome and systemic inflammation.

The “multiple-hit hypothesis” considers multiple mechanisms of liver damage in subjects with a genetic predisposition to develop FLD^10^. Insulin resistance, hyperlipidemia, and altered gut flora may be major factors for NAFLD or MAFLD development. These are factors that can also affect cataracts^11,12^, so it can be hypothesized that cataracts and fatty liver are closely related. The two diseases may be connected through various mechanisms; for example, patients with inflammatory bowel disease have a high prevalence of both fatty liver and cataract^13,14^.

However, the association between cataracts and FLD remains unclear. This study aimed to investigate the association between cataracts and FLD (NALFD and MAFLD) and determine whether FLD is an independent risk factor for cataracts. A nationwide Korean cross-sectional cohort was used, and well-known risk factors were adjusted for.

## Methods

### Dataset

We used a comprehensive cross-sectional health examination dataset based on the fifth Korean National Health and Nutrition Examination Survey (KNHANES V; available online at https://knhanes.kdca.go.kr/knhanes/eng/index.do) conducted between 2010 and 2011. The data collection protocol was approved by the Institutional Review Board of the Korean Centers for Disease Control and Prevention (no. 2010-02CON-21-C and 2011-02CON-06-C). Additionally, this study was exempted from ethical review by the Institutional Review Board of the Korean National Institute for Bioethics Policy because it used publicly accessible data. All participants signed forms consenting to the use of their health information. The KNHANES is a nationwide, population-based, cross-sectional survey conducted by the Division of Chronic Disease Surveillance of the Korea Centers for Disease Control and Prevention^15^. All participants were randomly selected using stratified sampling in which the following factors were considered: population, sex, age, regional area, and residential area. KNHANES comprised health records based on health interviews, health examinations, and nutrition surveys. Each participant provided health and socioeconomic information regarding age, household income, alcohol use, smoking status, hypertension, and diabetes^16^. Alcoholics were surveyed through a survey on whether they had experience with counseling for drinking problems. All participants underwent blood tests to evaluate their lipid profiles and liver enzyme levels after overnight fasting.

**Figure 1:**
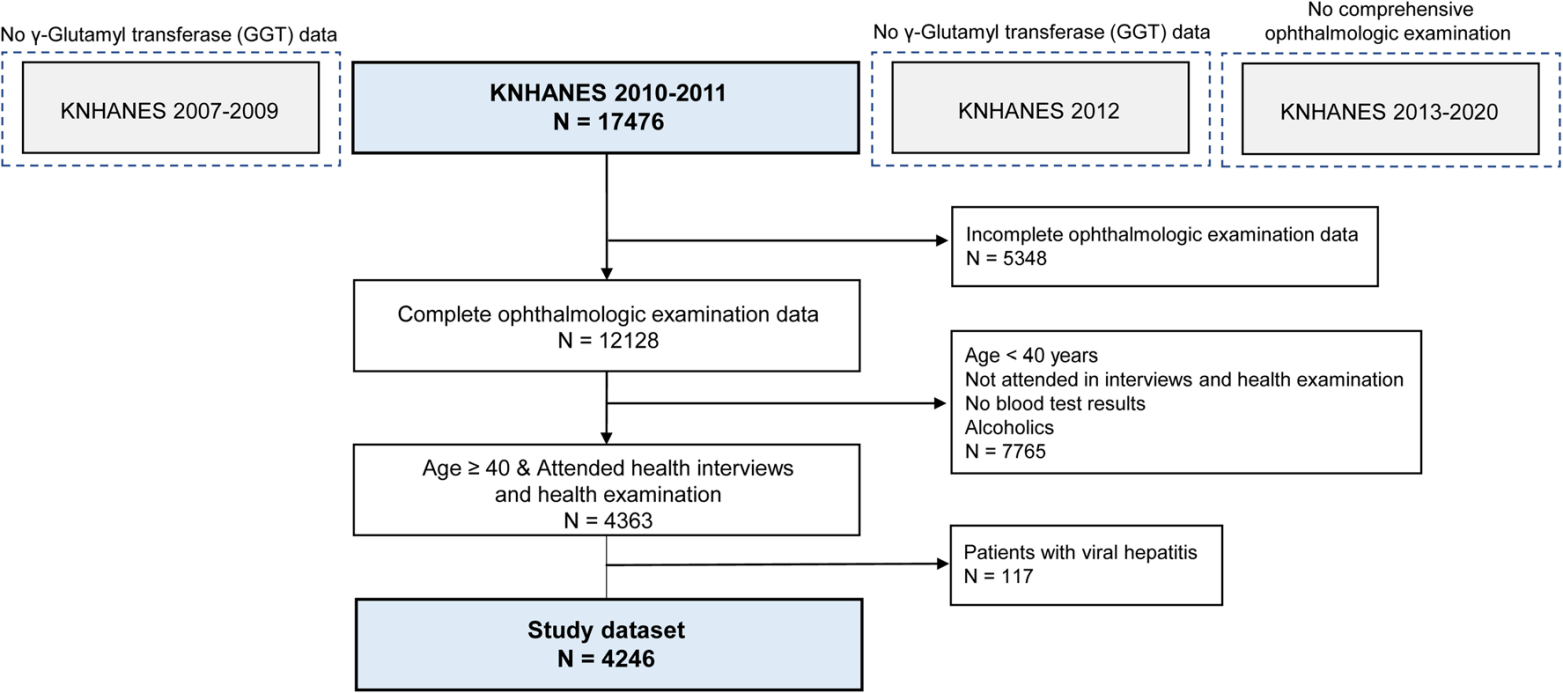
Study dataset flowchart in the Korean National Health and Nutrition Examination Survey (KNHANES).

In this study, Fig. 1 displays the inclusion and exclusion criteria. The KNHANES surveyed comprehensive eye examinations and liver enzyme, including gamma-glutamyl transferase (GGT), during 2010 and 2011. KNHANES datasets from difference time periods were excluded because of the lack of GGT or cataract evaluation. Initially, 17,476 participants were included in the KNHANES 2010–2011 dataset. We excluded 5,348 participants with incomplete ophthalmologic examinations, 7,765 participants aged (< 40 years) with incomplete survey data (interviews, health examinations, and blood tests) or alcoholics, and 117 participants with a history of viral hepatitis. Ultimately, the study included 4,246 participants.

### Definition of FLD

As the KNHANES did not conduct liver steatosis imaging tests, we adopted indirect indicators commonly utilized in large-scale epidemiological studies. For determining NAFLD and MAFLD, we referred to recent literature^17^. NAFLD was defined using previously validated formulas, such as the NAFLD fibrosis score and fibrosis-4 index (FIB4)^18,19^. Threshold values were applied using those described in a previous study^17^. MAFLD was defined using a previous formula based on the fatty liver index (FLI) and metabolic syndromes^20^. For MAFLD diagnosis, at least one evidence of metabolic syndrome must be present while satisfying an FLI ≥ 30^21^. The detailed formulas used to calculate the FLIs are described in the Supplementary Materials (See Supplementary Table S1). They were calculated based on a previous study using data from the KNHANES^22^. This score-based approach has been widely adopted in large epidemiological studies^23^, and showed very good predictability in a FLD-detection validation study^24^. According to the protocol of the previous study^17^, serum albumin was excluded from the NFS calculation due to insufficient data in KNHANES. NFS, FIB4, and FLI values calculated for analysis were coded into quartiles.

### Determining cataract status

Detailed protocols for determining cataract status in the KNHANES have been reported^11,25^. Eye examination quality was controlled by the Epidemiologic Survey Committee of the Korean Ophthalmologic Society (KOS). Participating ophthalmologists or residents were periodically trained by acting staff members of the National Epidemiologic Survey Committee of the KOS. The data quality and collection protocols were verified by the Korea Disease Control and Prevention Agency (KDCA).

In the KNHANES, a slit-lamp (Haag-Streit model BQ-900; Haag-Streit AG, Koeniz, Switzerland) examination was performed without pupil dilation^26^. The overall characteristics of the crystalline lens were examined to determine cataract subtype and severity level. Each lens layer was examined from the anterior to the posterior capsule using a focused slit lamp. During the examination, the Lens Opacities Classification System III (LOCS III) was used to evaluate cataract status^27^. It should be noted that the absence of pupil dilation made the diagnosis of peripheral lens opacities difficult and influenced the detection of cataracts in the peripheral lens. Investigators compared crystalline lens conditions with standard photographs of LOCS III images. Cataract subtypes were categorized as cortical (LOCS III score ≥ C2 for cortical opacity), nuclear (LOCS III score ≥ 4 for nuclear opalescence or nuclear color [NO4 or NC4]), and posterior subcapsular (LOCS III score ≥ P2 for posterior subcapsular opacity) cataracts (See Supplementary Figure S1)^25^. Pseudophakic eyes or eyes with a history of cataract surgery were also classified into the cataract group. For the subtype analysis, participants who had undergone cataract surgery in one eye were categorized as having pseudophakia. Those with primarily cortical cataracts in one eye and no other cataract types were classified as having pure cortical cataracts. Participants with nuclear cataracts were defined similarly. Participants who had minor subtypes of cataract (e.g., anterior and posterior subcapsular cataracts) or a combination of multiple subtypes simultaneously were classified as having other or mixed cataracts. The KNHANES has binarized cataract grading data into presence or absence of each subtype in the raw data according to the official research protocol established by the KOS and KDCA. These criteria and data have been utilized and validated in previous epidemiological studies^28,29^. Moreover, a similar protocol using the LOCS III system to determine cataracts has been verified in a well-known epidemiological study conducted in a Chinese population^30^.

### Statistical analysis

The variables according to the cataract or FLD groups were compared using the Wilcoxon rank-sum test for continuous data and χ^2^ test for categorical data. We used multinomial logistic regression to estimate the adjusted odds ratios (ORs) for each cataract type compared with the reference of participants without cataracts. Accordingly, logistic regression models determined the association between FLD (MALFD/NAFLD) and cataracts in the primary analysis while accounting for covariates. The adjusted ORs were calculated as follows: (1) model 1, wherein the ORs were adjusted for a priori covariates, including age, sex, and body mass index; and (2) model 2, wherein ORs were adjusted for the following covariates: age, sex, body mass index, current smoking status, alcohol consumption, hypertension, and diabetes mellitus. The KNHANES did not collect information on steroid use. Statistical significance was set at *P* < 0.05. Analyses used Statistical Package for the Social Sciences Statistics 25.0 (SPSS Inc., Chicago, IL, USA). *P* values in logistic regression model were adjusted using Benjamini–Hochberg false discovery rate (FDR) correction for multiple testing correction manner^31^. To address confounder collinearity (age, sex, body mass index, current smoking, alcohol consumption, hypertension, and diabetes mellitus), we assessed the correlation matrix, confirming no multicollinearity (all pairwise Pearson's correlation coefficients were < 0.5).

### Ethical approval

The data collection protocol was approved by the Institutional Review Board of the Korean Center for Disease Control and Prevention (No. 2010-02CON-21-C and 2011-02CON-06-C). Additionally, this study was exempt from ethical review by the Institutional Review Board of the Korean National Institute for Bioethics Policy because it used publicly accessible data.

### Informed consent

All participants signed forms that consented to the use of their health information during data collection (KNHANES V; available online at https://knhanes.kdca.go.kr/knhanes/eng/index.do).

## Results

Table 1 displays participant characteristics based on their NAFLD or MAFLD status. According to our definitions, 2007 participants (47.3%) had NAFLD, while 2050 (48.3%) had MAFLD. Participants with FLD exhibited higher body mass index (*P* < 0.001) and a greater prevalence of metabolic conditions, including hypertension and diabetes (*P* < 0.001). They also showed higher incidence of cataracts or pseudophakia (*P* < 0.001). Sex, smoking status, and alcohol intake demonstrated significant differences in relation to the FLD classification.

**Table 1:** Comparison of general and clinical characteristics among participants with and without fatty liver disease.

	NAFLD			MAFLD		
	Yes (N = 2007)	No (N = 2239)	*P*-value	Yes (N = 2050)	No (N = 2196)	*P*-value
Age (years)	57.3 ± 10.9	56.2 ± 11.0	0.001	57.2 ± 10.8	56.3 ± 11.1	0.005
Sex, female (%)	1162 (57.9)	1275 (56.9)	0.534	949 (46.3)	1488 (67.8)	< 0.001
Body mass index (kg/m^2^)	27.0 ± 2.9	22.6 ± 2.3	< 0.001	26.9 ± 2.9	22.63 ± 2.4	< 0.001
Household income			0.177			0.123
Very low (%)	498 (24.8)	498 (22.2)		495 (24.1)	501 (22.8)	
Low (%)	486 (24.2)	551 (24.6)		494 (24.1)	543 (24.7)	
Moderate (%)	517 (25.8)	577 (25.8)		550 (26.8)	544 (24.8)	
High (%)	506 (25.2)	613 (27.4)		511 (24.9)	608 (27.7)	
Current smoker (%)	366 (18.2)	365 (16.3)	0.103	461 (22.5)	270 (12.3)	< 0.001
Alcohol, ≥ 1 drink/week (%)	837 (41.7)	934 (41.7)	0.994	754 (36.8)	1017 (46.3)	< 0.001
Hypertension (%)	883 (44.0)	519 (23.2)	< 0.001	883 (43.1)	519 (23.6)	< 0.001
Diabetes mellitus (%)	464 (23.1)	102 (4.6)	< 0.001	388 (18.9)	178 (8.1)	< 0.001
Cataract or pseudophakia (%)	921 (45.9)	886 (39.6)	< 0.001	941 (45.9)	866 (39.4)	< 0.001
Cataract subtypes						
Cortical cataract (%)	476 (23.7)	468 (20.9)	0.029	485 (23.7)	459 (20.9)	0.032
Nuclear cataract (%)	175 (8.7)	155 (6.9)	0.029	180 (8.8)	150 (6.8)	0.019
Other or mixed cataracts (%)	132 (6.6)	150 (6.7)	0.902	145 (7.1)	137 (6.2)	0.295
Pseudophakia (%)	164 (8.2)	148 (6.6)	0.052	164 (3.9)	148 (3.5)	0.126

MAFLD: metabolic dysfunction-associated fatty liver disease, NAFLD: nonalcoholic fatty liver disease.

Table 2 presents clinical measurements related to FLD (variables for FLD scores) based on cataract presence. Participants with cataracts demonstrated higher waist circumference, fasting glucose, aspartate aminotransferase, and alkaline phosphatase, and lower total cholesterol, high-density lipoprotein cholesterol, alanine aminotransferase, and platelet count. Participants with cataracts had higher liver fibrosis scores, including NFS (*P* < 0.001), FIB4 (*P* < 0.001), and FLI (*P* = 0.001).

**Table 2:** Clinical measurements and fatty liver indices of participants with and without cataracts.

	Cataract (N = 1807)	No cataract (N = 2439)	*P*-value
Waist circumference (cm)	85.84 ± 9.56	83.43 ± 9.84	< 0.001
Laboratory variables			
Fasting glucose level (mg/dL)	105.40 ± 26.68	100.44 ± 24.89	< 0.001
Total cholesterol (mg/dL)	192.60 ± 38.46	196.02 ± 37.08	0.004
Triglyceride (mg/dL)	151.96 ± 119.66	146.17 ± 117.71	0.117
High-density lipoprotein cholesterol (mg/dL)	46.13 ± 11.07	48.06 ± 11.17	< 0.001
Low-density lipoprotein cholesterol (mg/dL)	116.06 ± 36.26	118.86 ± 34.31	0.010
Aspartate aminotransferase (IU/L)	24.29 ± 11.49	23.08 ± 10.92	0.001
Alanine aminotransferase (IU/L)	23.10 ± 15.73	24.18 ± 18.17	0.040
Alkaline phosphatase (IU/L)	249.45 ± 77.82	226.42 ± 68.03	< 0.001
γ-Glutamyl-transpeptidase (IU/L)	39.47 ± 64.83	38.40 ± 49.39	0.557
Platelet count (10^9^/L)	251.80 ± 60.77	256.34 ± 59.46	0.015
Liver fibrosis score			
NAFLD fibrosis score (NFS)	1.17 ± 1.12	0.39 ± 1.00	< 0.001
Fibrosis-4 index (FIB4)	1.45 ± 0.83	1.05 ± 0.62	< 0.001
Fatty liver index (FLI)	36.59 ± 25.56	33.80 ± 26.41	0.001

MAFLD: metabolic dysfunction-associated fatty liver disease, NAFLD: nonalcoholic fatty liver disease.

The results of the binary logistic regression models for cataract presence are presented in Table 3. In the models adjusted for related variables, cataract risk was associated with older age, hypertension, and diabetes. NAFLD was significantly associated with a higher OR for cataracts in both model 1 (OR = 1.50 [95% CI, 1.24–1.82], *P* < 0.001, Nagelkerke R^2^ = 0.407) and model 2 (OR = 1.23 [95% CI, 1.01–1.51], *P* = 0.048, Nagelkerke R^2^ = 0.425). However, after FDR correction, the adjusted *P* value for NAFLD did not reach statistical significance in the fully adjusted model (FDR-adjusted *P* = 0.058 in model 2). MAFLD was also significantly associated with a higher OR for cataracts in both model 1 (OR = 1.55 [95% CI, 1.27–1.89], *P* < 0.001, Nagelkerke R^2^ = 0.409) and model 2 (OR = 1.34 [95% CI, 1.10–1.64], *P* = 0.004, Nagelkerke R^2^ = 0.426). The Hosmer–Lemeshow test showed *P* > 0.050 in all logistic regression calculations. MAFLD consistently showed statistical significance after FDR correction (FDR-adjusted *P* < 0.001 in model 1; FDR-adjusted *P* = 0.006 in model 2).

**Table 3:** Logistic regression analysis of the association between cataracts and demographic or clinical factors.

Definition of FLD	Covariates	Model 1*			Model 2†		
		OR of cataract (95% CI)	*P*-value	Adjusted *P*-value‡	OR of cataract (95% CI)	*P*-value	Adjusted *P*-value‡
NAFLD	Age						
	≤ 60 years	1.00 (Reference)			1.00 (Reference)		
	> 60 and ≤ 70 years	8.04 (6.83–9.47)	< 0.001	< 0.001	6.89 (5.82–8.15)	< 0.001	< 0.001
	> 70 years	40.09 (30.16–53.29)	< 0.001	< 0.001	32.31 (24.20–43.13)	< 0.001	< 0.001
	Sex (female)	1.02 (0.88–1.19)	0.758	0.758	1.03 (0.87–1.22)	0.746	0.881
	Body mass index						
	≤ 20 kg/m^2^	1.00 (Reference)			1.00 (Reference)		
	> 20 and ≤ 25 kg/m^2^	0.56 (0.31–1.01)	0.561	0.063	0.52 (0.29–0.93)	0.029	0.041
	> 25 kg/m^2^	0.47 (0.25–0.86)	0.014	0.022	0.43 (0.23–0.79)	0.007	0.012
	Current smoker				0.99 (0.79–1.24)	0.949	0.949
	Alcohol, ≥ 1 drink/week				0.99 (0.85–1.16)	0.934	0.999
	Hypertension				1.74 (1.47–2.06)	< 0.001	< 0.001
	Diabetes mellitus				1.69 (1.33–2.15)	< 0.001	< 0.001
	NAFLD	1.50 (1.24–1.82)	< 0.001	< 0.001	1.23 (1.01–1.51)	0.048	0.058
MAFLD	Age						
	≤ 60 years	1.00 (Reference)			1.00 (Reference)		
	> 60 and ≤ 70 years	8.05 (6.83–9.48)	< 0.001	< 0.001	6.90 (5.83–8.17)	< 0.001	< 0.001
	> 70 years	39.98 (30.07–53.14)	< 0.001	< 0.001	32.15 (24.08–42.92)	< 0.001	< 0.001
	Sex (female)	1.13 (0.97–1.33)	0.116	0.116	1.09 (0.92–1.30)	0.313	0.369
	Body mass index						
	≤ 20 kg/m^2^	1.00 (Reference)			1.00 (Reference)		
	> 20 and ≤ 25 kg/m^2^	0.55 (0.30–0.99)	0.046	0.052	0.51 (0.28–0.91)	0.023	0.029
	> 25 kg/m^2^	0.45 (0.24–0.83)	0.011	0.015	0.40 (0.22–0.73)	0.003	0.005
	Current smoker				0.98 (0.79–1.22)	0.862	0.933
	Alcohol, ≥ 1 drink/week				1.01 (0.86–1.18)	0.900	0.900
	Hypertension				1.71 (1.45–2.03)	< 0.001	< 0.001
	Diabetes mellitus				1.74 (1.38–2.19)	< 0.001	< 0.001
	MAFLD	1.55 (1.27–1.89)	< 0.001	< 0.001	1.34 (1.10–1.64)	0.004	0.006

CI: confidence interval, MAFLD: metabolic dysfunction-associated fatty liver disease, NAFLD: nonalcoholic fatty liver disease, OR: odds ratio.

*Each OR analysis was adjusted for covariates including age, sex, and body mass index.

^†^Each analysis for the ORs was adjusted for the covariates including age, sex, body mass index, current smoking, alcohol consumption, hypertension, and diabetes mellitus.

^‡^*P* values were adjusted by Benjamini–Hochberg false discovery rate (FDR) correction for the multiple testing correction manner.

**Figure 2:**
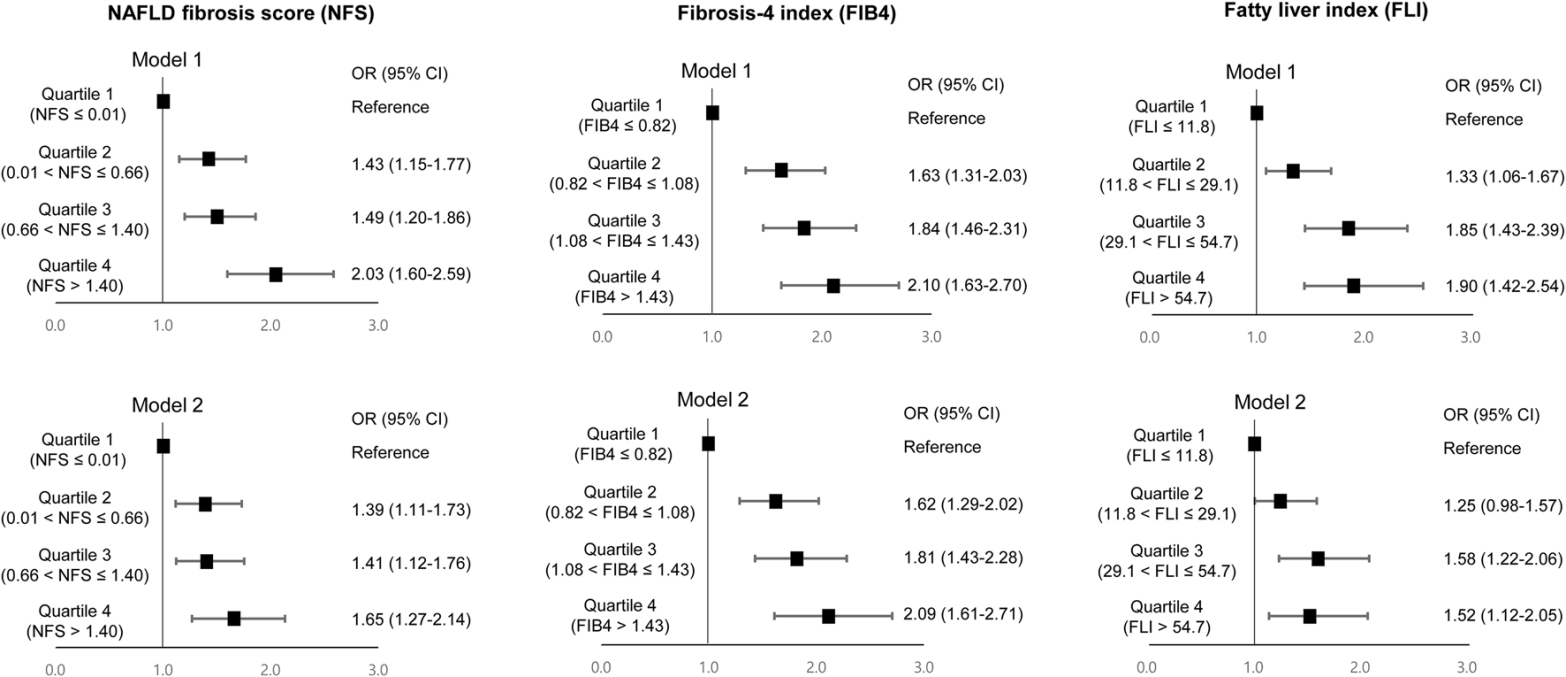
Plots for adjusted odds ratios (ORs) for cataract risk according to quartile of fatty liver indexes. Model 1 was adjusted for age, sex, and body mass index. Model 2 was adjusted for age, sex, body mass index, current smoker, alcohol consumption, hypertension, and diabetes mellitus. CI, confidence interval; FLI, fatty liver index; FIB4, fibrosis-4 index; NFS, nonalcoholic fatty liver disease fibrosis score; OR, odds ratio.

Figure 2 shows cataract risk according to FLD severity (quartile categorization of FLD indices). In the fully adjusted model (model 2, Nagelkerke R^2^ = 0.425), compared with the first quartile of NFS, the adjusted ORs were 1.39 (95% CI, 1.11–1.73, *P* = 0.002, FDR-adjusted *P* = 0.004), 1.41 (95% CI, 1.12–1.76, *P* = 0.002, FDR-adjusted *P* = 0.006), and 1.65 (95% CI, 1.27–2.14, *P* < 0.001, FDR-adjusted *P* < 0.001) in the second, third, and fourth quartiles, respectively (*P* value for linear trend < 0.001). Similarly, compared with the first quartile of FIB4 (model 2, Nagelkerke R^2^ = 0.434), the adjusted ORs were 1.62 (95% CI, 1.29–2.02, *P* < 0.001, FDR-adjusted *P* < 0.001), 1.81 (95% CI, 1.43–2.28, *P* < 0.001, FDR-adjusted *P* < 0.001), and 1.65 (95% CI, 1.61–2.71, *P* < 0.001, FDR-adjusted *P* < 0.001) in the second, third, and fourth quartiles, respectively (*P* value for linear trend < 0.001). FLI showed similar patterns with ORs of 1.25 (95% CI, 0.098–1.57, *P* = 0.078, FDR-adjusted *P* = 0.095), 1.58 (95% CI, 1.22–2.06, *P* = 0.001, FDR-adjusted *P* = 0.004), and 1.52 (95% CI, 1.12–2.05, *P* = 0.006, FDR-adjusted *P* = 0.010) in the second, third, and fourth quartiles, respectively (*P* value for linear trend = 0.003), compared with the first quartile (model 2, Nagelkerke R^2^ = 0.427).

The ORs for each cataract subtype were calculated to examine the association among subtypes and FLD. Table 4 shows the results of multinomial logistic regression analysis for each cataract subtype. In Model 1, NAFLD showed a statistically significant association with all cataract subtypes. After adjusting for all factors (model 2) with FDR correction, NAFLD was not associated with any cataract subtype (FDR-adjusted *P* = 0.062 for cortical cataracts, 0.155 for nuclear cataracts, 0.694 for other or mixed cataracts, and 0.151 for pseudophakia). MAFLD was significantly associated with all cataract subtypes in Model 1. In the fully adjusted model (Model 2), MAFLD consistently showed significant associations with cortical (FDR-adjusted *P* = 0.045), nuclear (FDR-adjusted *P* = 0.043), and other or mixed cataract (FDR-adjusted *P* = 0.027) subtypes. However, there was no significant associations with pseudophakia (FDR-adjusted *P* = 0.090).

**Table 4:** Results of multinomial logistic regression for analysis of the odds ratios for cataract subtype risk.

Definition of FLD	Cataract subtypes (target variable)	Model 1*			Model 2†		
		OR of cataract (95% CI)	*P*-value	Adjusted *P*-value‡	OR of cataract (95% CI)	*P*-value	Adjusted *P*-value‡
NAFLD	No cataract	1.00 (Reference)			1.00 (Reference)		
	Cortical cataract	1.68 (1.24–2.29)	0.001	0.002	1.42 (1.03–1.96)	0.034	0.062
	Nuclear cataract	1.42 (1.14–1.75)	0.001	0.003	1.21 (0.97–1.51)	0.099	0.155
	Other or mixed cataracts	1.49 (1.06–2.08)	0.022	0.031	1.09 (0.76–1.57)	0.636	0.694
	Pseudophakia	2.00 (1.37–2.91)	< 0.001	0.001	1.42 (0.95–2.12)	0.092	0.151
MAFLD	No cataract	1.00 (Reference)			1.00 (Reference)		
	Cortical cataract	1.66 (1.21–2.28)	0.002	0.002	1.44 (1.04–1.98)	0.026	0.045
	Nuclear cataract	1.45 (1.16–1.80)	0.001	0.002	1.29 (1.03–1.61)	0.027	0.043
	Other or mixed cataracts	1.86 (1.32–2.61)	< 0.001	0.001	1.55 (1.09–2.20)	0.014	0.027
	Pseudophakia	1.74 (1.19–2.55)	0.004	0.006	1.45 (0.98–2.14)	0.060	0.090

CI: confidence interval, FLD: fatty liver disease, MAFLD: metabolic dysfunction-associated fatty liver disease, NAFLD: nonalcoholic fatty liver disease, OR: odds ratio.

*Each OR analysis was adjusted for covariates including age, sex, and body mass index.

^†^Each analysis for the ORs was adjusted for the covariates including age, sex, body mass index, current smoking, alcohol consumption, hypertension, and diabetes mellitus.

^‡^*P* values were adjusted by Benjamini–Hochberg false discovery rate (FDR) correction for the multiple testing correction manner.

## Discussion

In this population-based cross-sectional study of South Korean adults, we found that MAFLD was significantly associated with cataracts after adjusting for general cataract risk factors, including age, diabetes, and hypertension. After adjusting for all the factors, NAFLD was found to not be associated with any cataract subtype. This indicates that MAFLD may be an independent cataract risk factor. Significant relationships were observed with all cataract subtypes; however, no significant relationships were reported for pseudophakia. Our analysis confirmed that overall cataract risk increased in participants with FLD. Furthermore, FLD severity (NFS, FIB4, and FLI) was associated with an increased cataract risk, with significant linear trends. To the best of our knowledge, this is the first study to evaluate the relationship between FLD and cataracts.

FLD is a common chronic liver disease and a global health concern^32^, with a prevalence of 25–40% worldwide^33^. As we excluded participants aged < 40 years, our study showed a similar prevalence, highlighting FLD as a significant issue in Korean society. Recently, FLD was recognized as an important pathological condition associated with liver inflammation and metabolic degeneration, and various health problems such as stroke, obstructive sleep apnea, atherosclerosis, heart failure, peripheral artery disease, and chronic kidney disease^34^. Because the eye is also an organ rich in blood vessels, it is also impacted FLD progression.

**Figure 3:**
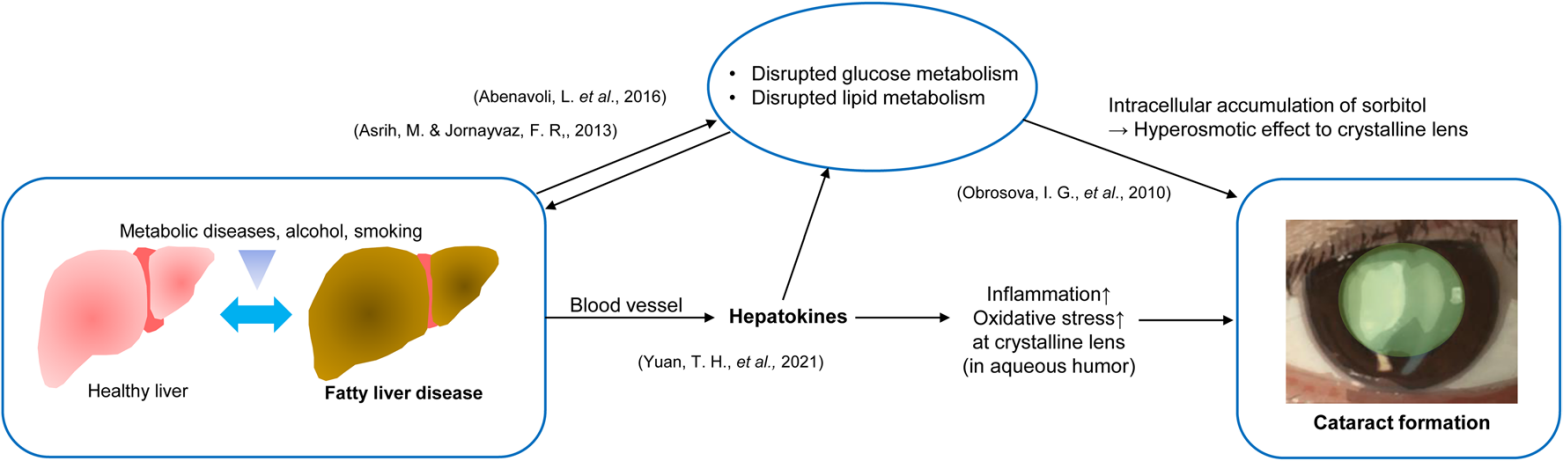
Schematic diagram of possible cataract pathogenesis in patients with fatty liver disease. FLD is a chronic inflammatory condition associated with type 2 diabetes and dyslipidemia^32,36^. In patients with diabetes, hyperglycemia accelerates sorbitol production in the crystalline lens^35^. Hepatokines can reach the eyes, while inflammation and oxidative stress resulting from FLD could further contribute to development of cataract^38^.

MAFLD exhibited a stronger relationship with cataracts compared with NAFLD, primarily because of its comprehensive inclusion of liver cirrhosis resulting from alcohol consumption and various metabolic diseases. Considering that cataract can be caused by various metabolic and inflammatory conditions, we hypothesized a link between FLD and cataract development. Figure 3 illustrates the potential pathogenesis of cataracts in patients with FLD, where both metabolic and inflammatory mediators of FLD may contribute to cataract development. NAFLD is a known risk factor for metabolic and cardiovascular diseases^23^, while MALFD, recently renamed to highlight the metabolic factors of FLD, demonstrated a similar association with other chronic diseases^21^. FLD is now recognized as a chronic inflammatory condition associated with type 2 diabetes and dyslipidemia^32^. Previous studies have focused on the relationship between diabetes and cataracts^1^, where the intracellular sorbitol accumulation in the crystalline lens leads to osmotic stress and liquefaction of lens fibers^35^. In patients with diabetes, hyperglycemia accelerates sorbitol production. Because the cytokines in FLD directly affect insulin resistance in the liver^32^, FLD could exacerbate diabetic cataract development. FLD is also closely associated with dyslipidemia^36^, and disruption in lipid metabolism caused by FLD may contribute to cataract development^37^. Furthermore, hepatokines, signaling proteins secreted by the liver, can reach the eyes^38^, while inflammation and oxidative stress resulting from FLD could further contribute to development of cataract.

Although the impact of diabetes on the eye has been extensively studied^39^, the association between liver diseases and ocular conditions remain unexplored. A recent deep learning-based study demonstrated the potential for detecting hepatobiliary diseases through slit-lamp and fundus photography images^40^. Although the prediction accuracy was not high, the study indicated a connection between eye and liver disorders, including liver cirrhosis, hepatitis, and NAFLD. A literature review highlighted the role of circulating hepatokines, such as fibroblast growth factor-21, hepatocyte growth factor, and angiopoietin-like proteins, in FLD, which can contribute to the development of pterygium, age-related macular degeneration, and diabetic retinopathy^38^. Disturbances in glucose, lipids, and amino acids may affect the eyes through various mechanisms^41^, although epidemiological evidence is currently lacking. This pathogenesis may also influence cataract development, as demonstrated in our large cross-sectional cohort study. Further investigations are needed to validate these potential mechanisms linking FLD and ophthalmic diseases.

One strength of our study is that we analyzed data from the KNHANES, a nationwide survey conducted on a large scare. Therefore, we produced representative results regarding the relationship between cataracts and FLD, with minimal selection bias. Another advantage is that we analyzed both NAFLD and MAFLD to investigate the influence of the broad spectrum of FLD, rather than specific conditions alone. We also studied the dose–response association between well-defined fatty liver indices, including NFS, FIB4, and FLI. Additionally, we adjusted for a wide range of cataract-related covariates to determine the independent association between FLD and cataract risk.

This study has several limitations. First, the cross-sectional nature of our study hinders us from establishing causality between FLD and cataracts. To confirm the associated, a longitudinal controlled research design is required. Second, this study was conducted in a single Asian country, raising uncertainty about the generatability of our findings to other countries or ethnic groups. Third, measurements of glucose levels, cholesterol profile, liver enzyme profile, and body mass index may vary depending on the timing of sample collection. Fourth, the lack of information on steroid use in the KNHANES dataset is another limitation. Because prolonged glucocorticoid use is a major risk factor for posterior subcapsular cataracts^42^, the absence of this information might have confounded our results. However, in this study, most patients had nuclear and cortical cataracts; therefore, the confounding effects of steroid use were estimated to be small. Fifth, the KNHANES did not record the detailed grades according to LOCS III. The cataract grading based on LOCS III might be subjective and inconsistent without pupil dilation. To overcome this problem, the KOS established clear criteria for each cataract subtype and collected data with binary outcomes. The KOS has appropriately trained investigators to effectively collect and analyze data for large epidemiological studies.

## Conclusion

This study revealed that FLD was significantly associated with a higher cataract risk in Korean adults. After adjustment for well-known cataract risk factors, MAFLD exhibited a strong association with cataracts. Our comprehensive analysis suggests that FLD may serve as an independent risk factor for cataracts. These findings underscore the importance of addressing modifiable risk factors, such as diabetes, hypertension, and FLD in the prevention of cataracts. Further larger-scale, longitudinal, and controlled studies are required to validate the impact of FLD on cataracts.

### Supplementary Information

Supplementary Information.

## Data Availability

This study is based on the fifth Korean National Health and Nutrition Examination Survey (KNHANES V; available online at https://knhanes.kdca.go.kr/knhanes/eng/index.do) conducted between 2010 and 2011.

## Abbreviations

CI: Confidence interval
KNHANES: Korean National Health and Nutrition Examination Survey
KOS: Korean Ophthalmologic Society
FDR: False discovery rate
FIB4: Fibrosis-4 index
FLD: Fatty liver disease
FLI: Fatty liver index
GGT: Gamma-glutamyl transferase
LOCS: Lens Opacities Classification System
MAFLD: Metabolic dysfunction-associated fatty liver disease
NAFLD: Nonalcoholic fatty liver disease
NFS: Nonalcoholic fatty liver disease fibrosis score
OR: Odds ratio
