# Supplementary material for: Association between cataract and fatty liver diseases from a nationwide cross-sectional study in South Korea
Source: Sci Rep. 2024 Jan 2;14:77. doi: 10.1038/s41598-023-50582-7 (PMC10761897; doi:10.1038/s41598-023-50582-7)
Supplement: Supplementary file 1 — Supplementary Information. [file 41598_2023_50582_MOESM1_ESM.pdf]

Association between Cataract and Fatty Liver Diseases: A Nationwide Cross-Sectional Study from South Korea

Supplementary Materials

Supplementary Table S1. Definitions of fatty liver indexes including fatty liver index (FLI), NAFLD fibrosis score (NFS), and fibrosis-4 index (FIB4) for this study.

Supplementary Figure S1. Cataract grading system in the KNHANES.

Supplementary Table S1. Definitions of fatty liver indexes including fatty liver index (FLI), NAFLD fibrosis score (NFS), and fibrosis-4 index (FIB4) for this study.

Index	Formula
FLI	Exp(0.953 × ln(TG (mg/dL)) + 0.139 × BMI (kg/m2) + 0.718 × ln(GGT (IU/L)) + 0.053 × WC (cm)-15.745)/(1 + Exp(0.953 × ln(TG (mg/dL)) + 0.139 × BMI (kg/m2) + 0.718 × ln(GGT (IU/L)) + 0.053 × WC (cm)-15.745)) × 100
NFS*	-1.675 + 0.037 x age (years) + 0.094 x BMI (kg/m2) + 1.13 x impaired fasting glucose or diabetes (yes=1, no=0) + 0.99 x AST/ALT ratio - 0.013 x platelet count (×10^9/L) - 0.66 x serum albumin [g/dL]
FIB4	age (years) x AST (IU/L) / (platelet count (×10^9/L) x (ALT (IU/L))$^{1/2}$

ALT, Alanine aminotransferase; AST, Aspartate aminotransferase; BMI, body mass index; GGT, γ-Glutamyl-transpeptidase; TG, Triglyceride.

* Serum albumin was not used to calculate NFS due to lack of data in the KNHANES.

(Questionnaire)

Test for three poles (over 19 years of age)		
4.1. Cataract examination		
Separation	the right eye	the left bank
presence or absence of cataracts	**(E_Tr_y)**	**(E_Tl_y)**
an artificial lens	☐	☐
an indefinite fixed plan	☐	☐
There is no cataract.	☐	☐
to have cataracts	☐	☐
Classify if cataracts are present	**(E_Tr_c)**	**(E_Tl_c)**
1.Cortical type	☐	☐
2.Nuclear type	☐	☐
3.Anterior (sub)capsular type	☐	☐
4.Posterior subcapsular type	☐	☐
5.Mixed type	☐	☐

Supplementary Figure S1. Cataract grading system in the KNHANES.
